# The Mansion House Hospital Sunday Conversazione

**Published:** 1894-06-16

**Authors:** 


### THE MANSION HOUSE HOSPITAL SUNDAY CONVERSAZIONE.

Seldom have the beautiful reception rooms at the Mansion House contained a more iuteresting and interested assembly than they did on Friday, June 7th. The courteous invitations issued by the Lord Mayor were cordially accepted by those who know, as well as by those who wished to learn, all about hospitals. The guests who streamed into the hall included many of the best known hospital authorities, besides many clergy and others whose keen appreciation of all the details and difficulties of the work which the Hospital Sunday Fund aims at advancing, was peculiarly evident. Some excellent music was rendered by the City Police Band at the beginning of the evening, and was followed by charming songs sung by Miss Chamberlain, Miss Kate Cone, Mr. Beauchamp, and Mr. Grice. These were given at intervals both before and after the chief feature of the evening was introduced. This consisted of an exhibition in the Egyptian Hall of a series of "Living Pictures from the Hospitals," explained by Mr. Burdett, who had devoted much time to the preparation of this new and most attractive exhibition. The pictures displayed were so beautiful and artistic that it goes without saying that they must be costly productions, and no pains have evidently been spared to make the series of scenes complete and unique impressions of the hospitals of the day. It is to be hoped that the exhibition will prove of incalculable value to the cause of these institutions, and that these beautiful lime-lighted pictures may be shown on many future occasions to other audiences as intensely interested as the one privileged to enjoy them on "the first day," or rather first night.

After a few words of introduction Mr. Burdett showed a narrow street where the patients live when at home. The small houses, with the inevitable gin palace at the corner of the street, shelter far larger families than would be suspected from their exteriors. If the front of one of these dwellings could be displaced for a moment, such crowded hives of humanity would be disclosed as would cause dismay to the mind of sanitary or other inspectors.

From this street, with its narrow pavement and stony roadway, the rapidly-dissolving view changed to the hospital itself, and there the patients were seen both without and within the out-patient department. A succession of pictures brought with startling distinctness every smallest detail to the notice of the spectators—the men "waiting to see the doctor," and then the women seated on the benches in their special department.

A picture of the out-patient refreshment stall excited some attention, and Mr. Burdett remarked that "waiting to see the doctor" was by no means the poor man's prerogative, it being an experience which he shared with the rich who waited, often for hours, to take their turn for admission to a popular West-end physician's consulting room. One of the slides, entitled "With the Dentist," represented a long room with a vista of dentist's chairs, each occupied by a patient, some seventy altogether, and all receiving attention at the hands of the dentists and their assistants. A succession of wonderfully life-like pictures showed a street accident, the ambulance litter, the porters (a famous group of competent men), the casualty room, and the lift with the patient in it, and finally his admittance to the ward itself. Life in the wards was next depicted in a number of excellent pictures from the early bed-making to the evening service. The doctor's visit and the dinner hour were capitally shown, and so was the afternoon airing of a group of patients who had been carried, beds and all, into a balcony overlooking the Thames.

It is not possible to enumerate all the subjects which rapidly followed each other. Some of the children were so charming that they were greeted with loud applause. So faithfully were characteristic curves and attitudes reproduced that the exhibition of these life-like little figures became, as it were, a [illegible]velation of modern art. The whole days, and almost the whole life history of the suffering poor appeared to be brought before the attentive and appreciative audience which thronged the Egyptian Hall.

One of the quaintest, if not exactly the pleasantest, part of the display was that of the contrasting operation rooms of past and present times. The latter was well shown as well as the administration of the anæsthetic. The barbarous proceedings of earlier days were given by means of photographs taken from curious old engravings, one of which showed the terrible manner in which an amputation was conducted without chloroform. Parkwood Convalescent Home, faithfully depicted in a series of charming pictures, appeared as an ideal health resort, surrounded by breezy commons, and with every internal and external arrangement which could conduce to the welfare of the patients. Fever and small-pox hospitals received due attention, and a party of youngsters at breakfast, convalescent from scarlet fever, was a perfectly charming specimen of the photographer's art. Under the descriptive title of "The Nursing Department" a number of excellent groups and single figures of sisters, staff nurses and probationers from different hospitals, were shown, and many amused or admiring remarks were heard as the guests present recognised familiar faces and uniforms.

When Mr. Burdett had concluded his remarks, the Lord Mayor proposed a vote of thanks to him, which Sir Sydney Waterlow seconded, and the spectators cordially endorsed. Amongst the guests present were Dr. Adler, Cardinal Vaughan, Canon Ingram, Mr. A. L. Cohen, the Dean of St. Paul's and Miss Gregory, Admiral and Lady Egerton, Canon and Mrs. Barker, Lord Sandhurst, the Archdeacon of Middlesex, Prebendary Whittington, Dr. and Mrs. Sansom, Alderman and Mrs. Dimsdale, Prebendary Wilmot, Miss Gordon, Miss Monk, Miss R. Paget, Miss A. Hughes, Mrs. Burdett, Miss Hicks, and many others.

